# Research on Disability Grading Based on ICF Functional Framework: Empirical Evidence From Zhejiang Province, China

**DOI:** 10.3389/fpubh.2021.616180

**Published:** 2021-05-11

**Authors:** Huan Liu

**Affiliations:** School of Public Administration, Zhejiang University of Finance and Economics, Hangzhou, China

**Keywords:** disability, multi-state, grading, multi-dimensional, transition probability

## Abstract

Through assignment method, the total score of disability in multiple dimensions is obtained, and it is divided into five functional states—severe disability, partial disability, moderate disability, mild disability, and health—according to the score, and the probability of death is constructed. Using the Chinese Longitudinal Healthy Longevity Survey (CLHLS) database tracking survey data, by constructing a multistate transition probability matrix, the empirical calculation of the multistate disability transfer probability, with the help of the sixth national census data, we estimated maintenance time of each state, life expectancy, etc. The results show that the 3 year transfer probability of the initial healthy elderly is the highest, and the mortality rate is also the lowest. It can be found that the disability state transition probability measurement based on the data is more accurate than the model estimation; the disability scale and life expectancy estimated based on the multistate transition probability matrix are more reliable.

## Introduction

In the irreversible trend of rapid aging and aging of population, research on “aging health” has become an important part of the national development strategy in the world. In order to disperse the risk of huge care expenditure for the elderly, almost all developed countries have established public care systems since the 1960s. Among them, Nordic countries mostly provide public long-term care services, while Germany, Japan, South Korea, and other countries have successively established long-term care social insurance systems to cope with the aging population. In order to change the old-age security system which only paid attention to the economic security of the elderly to pay equal attention to economic security and service security, and achieved better economic and social benefits, the government and society of China have also focused on the issues of life care and economic security for the elderly who are old and frail and unable to take care of themselves. In April 2019, the general office of the State Council issued the opinions on promoting the development of elderly care services [(2019) No. 5]. The document defines the basic elements of “establishing and improving the long-term care service system,” such as standardizing the long-term care service items, standards, and quality evaluation and proposing to improve the professional long-term care service system linking home, community, and institutions. Among them, grading care and treatment payment are the most important parts of long-term care social insurance, and grading care is an important premise of long-term care service supply, the basis of treatment payment, and the key guarantee of long-term care service supply quality. However, combined with the existing practice and theoretical research, it is found that there are some problems in the current grading care and service supply; for e.g., service items are not unified, and service charges are not unified. Therefore, the study of disability grading is an important premise to promote the healthy development of long-term care insurance, which has important theoretical and practical significance.

## Literature Review

Combining the construction and pilot research of long-term care insurance systems in typical countries and regions at home and abroad, based on the perspective of long-term care insurance system construction, the main contents of long-term care insurance research can be summarized into two aspects, namely, the financing method of long-term care insurance and the payment scheme of long-term care insurance. From a theoretical perspective, disability assessment of the subject of long-term care insurance is an important basis for the implementation of the system, and also a reference for service supply. Therefore, the core of this paper is to sort out the existing literature from the disability grading and disability object scale, which is an important basis of the top-level design of long-term care insurance system.

From the perspective of countries practicing the long-term care insurance system, the demand screening of long-term care insurance is an important part of the implementation of the system. It is an important way to match the supply and demand of long-term care insurance and is the key to improving the supply effectiveness of the long-term care insurance system. The basis of demand screening is the assessment of disabled objects and the setting of their grading standards. Combined with the existing research, it can be found that there are relatively few researches on the grading of disabled objects, and most of them are based on practical analysis. As an important part of disability scale measurement, the transition probability of disability state is the premise and foundation to promoting demand assessment and will help to plan the medical and social services needed in a timely manner (1–3) and to reduce the burden on the elderly and their families who cannot take care of themselves (4, 5). First of all, regarding the evaluation indicators of incapacitated subjects (6–8), most studies use single-dimensional scales for measurement, such as the commonly used Barthel, Lawton, and Brody instrumental daily activity scales and the Katz Scale, and the international function/disability and health-grading ICF and InterRAI evaluation systems which are more commonly used in OECD countries (9, 10). There are also studies based on activities of daily living (ADL) and instrumental activities of daily living (IADL) (11–14). Secondly, in most studies, the macro-simulation prediction method and micro-simulation prediction method are used to study the transition probability of failure state. Moreover, due to the complexity, diversity, and dynamic characteristics of changes in health status of disability subjects, scholars often use multi-period panel data to study the dynamics of elderly health status from multiple dimensions. Evolution predicts the incidence of disability and its transfer. Among them, the first is the use of macroscopic simulation prediction methods, such as the European Economic Policy Committee's series of “Age Reports” (15). Moreover, because accurate and reliable microscopic data are difficult to obtain, some studies have therefore adopted this method (16, 17). The second is the microscopic simulation prediction method. Rickayzen and Walsh (18) first used the Markov method to predict the disability transition (19); since then, some studies have further applied and demonstrated the Markov method (20–22). Worrall and Chaussalet proposed that this method has a loss when micro data is available based on the comparative advantage of energy object measurement (23).

At present, a single-dimension scale is widely used in disability grading research (4). The main reason is that the single-dimension scale is becoming more and more perfect and has high reliability (24–28). As for the main grading research methods, the micro-simulation prediction method is more and more applied due to its higher prediction accuracy. However, existing studies still ignore the importance of disability grading from a multidimensional perspective, such as the coexistence of disability and dementia, and it is difficult to subjectively define that disability is more serious than dementia. Accurate measurement of the disabled elderly is an important basis for the implementation of long-term care social insurance and is the key to effectively measuring the demand and cost of long-term care insurance. However, most of the current studies focus on the traditional single-dimension measurement. Relatively speaking, the use of research based on multidimensional measurement indicators is relatively insufficient. Therefore, strengthening the research and application of the multidimensional disability assessment scale is the key to long-term care insurance in the future (29, 30). Based on the existing single-dimension disability index and ICF theoretical framework, this study will start with the construction of the multidimensional disability measurement index system and take Zhejiang Province as an example to explore the scale of the disabled population and the disability grading standard.

The innovations of this study are as follows: firstly, based on the basic theory of ICF, we construct a multidimensional disability assessment index system, estimate the weight of each dimension according to the macro data, and test the effectiveness of the multidimensional index system by constructing the death probability model and multistate transition probability model. Second, after the comprehensive evaluation of the existing disability assessment methods, based on the micro perspective, the 3 year Markov chain is constructed, and the non-parametric method is used to ensure the reliability of the multistate transition probability and improve the accuracy of the disability population size and multistate expected lifespan assessment.

## Research Methods and Data

### Research Methods

#### Disability Grading

Reviewing the existing methods for defining disability levels at home and abroad, based on the ICF theory framework's interactive influence on the concept of the disability from multiple dimensions such as restricted activity, physical and structural damage, limited participation, and health problems (disorders or diseases), we discuss the energy index system, set the functional areas of the elderly, and try to establish a multidimensional disability measurement index system based on the multifunctional field. The International grading of functioning, disability, and Heath (ICF) is an international functional grading developed by the World Health Organization. Its core is a disease management tool based on the modern medical model, which includes multiple dimensions such as physical function, disability, and health problems (31).

Firstly, the distribution characteristics of the disabled elderly are described and the grading standards of the disabled elderly are discussed. The disability grading is divided according to the international general grading fitting tool and the functional area of the project to evaluate the elderly, and a percentage system is implemented. The higher the score, the higher the nursing level. Secondly, the effectiveness test of disability grading uses the logit decision model to discuss the impact of disability index selection on the disability grading and uses the ordered logit model to analyze restricted activity, physical and structural damage, limited participation, health problems, and hierarchical relationship with the elderly.

#### Disability Transition Probability and Transition Intensity

Regarding the research on the transfer of disability of the elderly, the current academic circles mainly use macroscopic simulation prediction methods or microscopic simulation prediction methods. After comparing the two forecasting methods, macro and micro, it is found that the macro forecasting method is limited by the selection and assumptions of many key factors. When the data is available, the forecast model based on micro data is relatively dominant, but there is room for improvement in its application. For example, the construction of the transfer probability matrix directly borrows the foreign transfer probability or the simple chain-to-loop adjustment method with low accuracy, which cannot meet the design requirements of the insurance system. In the regression simulation method, the choice of explanatory variables in the model is diverse and subjective due to the complex and diverse health conditions. Therefore, when the data is available, the transition probability is constructed based on the data itself to better observe the dynamic evolution of the health status of the elderly, so the multistate Markov model is more suitable. The multistate Markov process is a family of random variables {S(t), tT} that depends on changing parameters. It is assumed that the disabled state of the elderly at time t is known and determined, at the time ζ (ζ > t) the old man's state and Before this time, the state is irrelevant, and a Markov process is formed; if the transition probability is related to the starting state and the arrival state, and is not related to the starting time, the Markov chain is said to be time-homogeneous. Among them, the set T of all values of the variable parameter t is the parameter space, and the value of S(t) constitutes the state space of the random process.

The research method of this paper is based on the ICF framework, combined with relevant population prediction methods, taking the data of the disabled population in China as the core, trying to explore the disability grading and its effectiveness. Then, the size of the disabled population and its long-term care insurance demand are accurately estimated, in order to provide reliable empirical support for the effective implementation of long-term care insurance. At the same time, the rationality of the ICF framework design is tested through the effectiveness of disability grading, which provides a perfect practical basis for the ICF theoretical framework.

### Data

#### Data Sources

The data in this article is mainly derived from the survey data of the Chinese Elderly Health and Longevity Influencing Factors (CLHLS) survey in 2011 and 2014. The survey scope of the CLHLS database covers 23 provinces, autonomous regions, and cities in China. The survey objects are mainly the elderly aged 65 and above and their adult children aged 35–64. The contents of the survey included the basic situation of the elderly and their families, socioeconomic background and family structure, economic source and economic status, self-assessment of health and quality of life, cognitive function, personality and psychological characteristics, daily activities, lifestyle, life care, disease treatment, and medical expenses. Therefore, on the whole, the database can reflect the reality of China's population and is suitable for research as basic data.

#### Data Processing

The existing data of the Chinese Longitudinal Healthy Longevity Survey (CLHLS) are combined to screen the samples. The specific processing is as follows: The core explained variables of the article mainly include disability and death. First, the death variable is traced according to the death samples from 2011 to 2014. According to the year of death, the individual who died is defined as 1, and the other is defined as 0. Second, based on the above analysis, based on the ICF functional structure framework, we try to select the corresponding secondary indicators from the four dimensions of restricted activity, physical function and structural damage, restricted participation, and health for measurement. In the specific processing, the integral system is used to measure the overall functional status. Among them, the activity-restricted dimension has a function score range of 0–50 points. In the physical function and structural damage dimension, the range of scores is 0 ~ 60 points. In the participation restricted dimension, the score range is 0 ~ 30 points. In the health dimension, the score range is 0 ~ 100 points.

In terms of disability grading, based on previous studies, it is calculated from multiple dimensions and graded according to the total score, as follows: the total score is above 200 points and marked as healthy, which is recorded as 5; 150 ~ 200 points are for mild disability—yes, recorded as 4; 100 ~ 150 is classified as moderate disability, recorded as 3; 50 ~ 100 is classified as severely disabled, recorded as 2; and 50 points or less is severely disabled, recorded as 1.

Second are the core explanatory variables: in terms of urban and rural indicators, merge cities and townships into urban and rural areas are recorded as 1, while rural areas are marked as 0; for marital indicators, widowed is marked as 1, and other married, unmarried, etc., are marked as 0; in terms of aspect in gender, males are marked as 1 and females as 0; and in the state of death, 1 is marked as death and other marks are 0.

#### Descriptive Statistics of Core Variables

The core explanatory variables mainly include disability grading status and mortality; the core explanatory variables include health status, physical limitation, and physical damage status, which correspond to the four dimensions of disability grading. Since the disability state is obtained from multiple dimensions, a substitute test is therefore conducted based on the investigation of the overall problems of each dimension in the sample. Among them, the health status is based on the sample overall health self-assessment agent, that is, choose “how do you evaluate your health?,” so as to reflect the health dimension; the physical restriction degree selected is “at least in the last 6 months, are you because of health Problems and unable to carry out normal activities?” The agent reflects his limited activity and limited participation dimensions; his physical function is based on the direct observation of the interviewee in the sample, reflecting his physical function and structural damage dimensions—happening. The specific index definition and sample size are shown in Table 1. The statistical sample is the total sample size of 2 years.

**Table 1:** Descriptive statistics of core variables.

**Variable**	**Definition**	**Percentage (%)**	**Frequency**
Disability grading	Severe disability = 1	4.7	904
	Overweight disability = 2	5.3	1,020
	Moderate disability = 3	9	1,753
	Mild disability = 4	13.9	2,706
	Health = 5	67.1	13,031
Mortality rate	Death = 1	21.1	4,097
	Survival = 0	78.9	15,317
Health status	Very poor = 1	1.6	227
	Poor = 2	15.5	2,238
	Normal = 3	38.5	5,560
	Good = 4	34.7	5,012
	Very good = 5	9.7	1,399
Degree of physical restraint	Severely restricted = 1	13.9	2,160
	Restricted = 2	23.5	3,642
	Completely unrestricted = 3	62.6	9,706
Physical function	Severe illness (riding in bed) = 1	2.5	390
	Moderate illness (moderately severe illness) = 2	15.9	2,458
	Relative health (with minor illness) = 3	59.9	9,266
	Health (without illness) = 4	21.7	3,348

## Empirical Test Results and Analysis

### Three-Year Probability of Death

According to the previous analysis, the article first examines the multidimensional index system of disability. Specifically, we construct a 3 year mortality probability model and a disability state transition probability model to examine whether multidimensional selection is effective by age; the test results are shown in Tables 2, 3, respectively. Among them, the results in Table 2 show that for the elderly below 95+ of age, males and widowed elderly have a significantly higher probability of death, and as age increases, the impact of this significant trend is decreasing, as shown in the table. The influence coefficients of different age groups are decreasing, and after 95+ years, these factors no longer have a significant impact on the mortality of the elderly; urban and rural factors have no significant impact on the mortality of the elderly. In terms of multidimensional disability indicators, for the elderly below 95+ of age, the physical function and the degree of physical restriction significantly affect the mortality rate, while the health status only has a significant effect on the elderly aged 75–85 years. The above results show that when the elderly are in a worse physical condition, the elderly have a higher mortality rate, but as the age increases, this effect is also reduced. For example, the effect of physical restriction on the 65–74 year-old elderly reaches 1.395, and it dropped to 0.624 for the elderly in the 95+ age group. In addition, in the multidimensional disability state, the effect of physical function on the death rate of the elderly reached the maximum, followed by the degree of physical restriction, and the lowest was the healthy state.

**Table 2:** Logit death probability model.

**Variable**	**The explained variable: the probability of death of the elderly in different age groups**			
	**Age group 65 **~** 74**	**Age group 75 **~** 85**	**Age group 85 **~** 95**	**Age group 95+**
	**(1)**	**(2)**	**(3)**	**(4)**
Physical function	−2.180^**^	−1.021^***^	−1.003^***^	−0.570
	(1.063)	(0.261)	(0.216)	(0.413)
Degree of physical restraint	−1.395^*^	−0.838^***^	−0.647^***^	−0.624^*^
	(0.718)	(0.221)	(0.171)	(0.333)
Health status	−0.864	−0.378^**^	−0.159	−0.134
	(0.551)	(0.191)	(0.148)	(0.301)
Town = 1	1.078	0.135	0.233	0.411
	(0.871)	(0.299)	(0.237)	(0.484)
Male = 1	1.820^*^	1.465^***^	1.002^***^	0.468
	(1.024)	(0.354)	(0.261)	(0.543)
Widowed = 1	2.119^**^	0.845^**^	0.653^**^	0.152
	(1.017)	(0.332)	(0.293)	(0.841)
/lnsig2u	3.652^***^	3.063^***^	3.273^***^	6.170^***^
	(0.131)	(0.0680)	(0.0749)	(0.0717)
Log likelihood	−188.0762	−574.4192	−1,133.8064	−1,048.6919
LR test	16.20 (*p* = 0.00)	46.42 (*p* = 0.00)	136.48 (*p* = 0.00)	277.86 (*p* = 0.00)
Observations	2,890	4,377	4,079	2,909

*The random effect of the panel logit model is used for testing here. The significance of the LR test results indicates that the random-effect regression is effective; in addition, the standard errors are in parentheses, where*

* *p < 0.1*,

** *p < 0.05*,

*** *p < 0.01*.

**Table 3:** Logit disability state transition probability model.

**Variable**	**Explained variable: disability rating of the elderly in different age groups**			
	**Age group 65 **~** 74**	**Age group 75 **~** 85**	**Age group 85 **~** 95**	**Age group 95+**
	**(1)**	**(2)**	**(3)**	**(4)**
Physical function	1.665^***^	1.462^***^	1.430^***^	1.028^***^
	(0.219)	(0.108)	(0.0871)	(0.0795)
Degree of physical restraint	1.644^***^	1.499^***^	1.554^***^	1.331^***^
	(0.189)	(0.0933)	(0.0754)	(0.0714)
Health status	0.614^***^	0.338^***^	0.0637	0.174^***^
	(0.145)	(0.0674)	(0.0518)	(0.0535)
Town = 1	−0.176	−0.0597	−0.0406	−0.140
	(0.215)	(0.105)	(0.0827)	(0.0858)
Male = 1	0.253	0.582^***^	0.715^***^	0.805^***^
	(0.235)	(0.120)	(0.0951)	(0.106)
Widowed = 1	−0.481^*^	−0.392^***^	−0.351^***^	−0.629^***^
	(0.250)	(0.116)	(0.104)	(0.157)
Threshold parameter 1	1.807^***^	1.321^***^	2.166^***^	2.006^***^
	(0.689)	(0.354)	(0.259)	(0.273)
Threshold parameter 2	3.398^***^	3.389^***^	4.028^***^	4.074^***^
	(0.627)	(0.305)	(0.264)	(0.295)
Threshold parameter 3	5.118^***^	5.281^***^	6.149^***^	6.057^***^
	(0.660)	(0.334)	(0.303)	(0.334)
Threshold parameter 4	7.291^***^	7.416^***^	8.333^***^	8.174^***^
	(0.750)	(0.392)	(0.356)	(0.386)
sigma2_u	3.167^***^	1.629^***^	1.652^***^	1.356^***^
	(1.023)	(0.382)	(0.300)	(0.272)
Log likelihood	−680.9276	−2,277.7828	−3,934.0044	−3,726.4067
LR test	24.58 (*p* = 0.00)	37.94 (*p* = 0.00)	64.05 (*p* = 0.00)	48.47 (*p* = 0.00)
Observations	2,890	4,377	4,079	2,909

*The random effect of the panel ordered logit model is selected for testing here. The significance of the LR test result indicates that the random-effect regression is effective; in addition, the standard errors are in parentheses, where*

* *p < 0.1*,

** *p < 0.05 and,*

*** *p < 0.01*.

### Three-Year Disability Transition Probability

According to the death probability models of the elderly at different ages, the probability of transition of the disabled state of the elderly is further modeled and tested. The results are shown in Table 3. The significant results of the threshold parameter test indicate that the five divided categories of the disabled state are effective. Compared with the probability of death, it is found that urban and rural factors also have no significant effect on the probability of the transfer of disability of the elderly, but gender still has a significant effect on the transfer of the state of disability of the elderly, and its influence coefficient on the elderly over 75 is greater. With the increase of age, the probability of transition of the disability state is more affected by gender; widowhood is opposite to the death probability model, showing a phenomenon of a negative reduction in the probability of transition of the disability state of the elderly. In terms of multiple dimensions of disability, the results in Table 3 show that changes in the physical function, physical restriction, and health status of the elderly during the 3 years have a significant positive effect on the probability of transition of the disability. The impact of the state transition probability is decreasing; that is, the mortality rate is increasing accordingly. It further explains the effectiveness of the construction of the multidimensional disability grading index in this paper, which can effectively reflect the disability status of the elderly.

### Calculation of the Disability State Transition Probability Matrix

**Figure 1:**
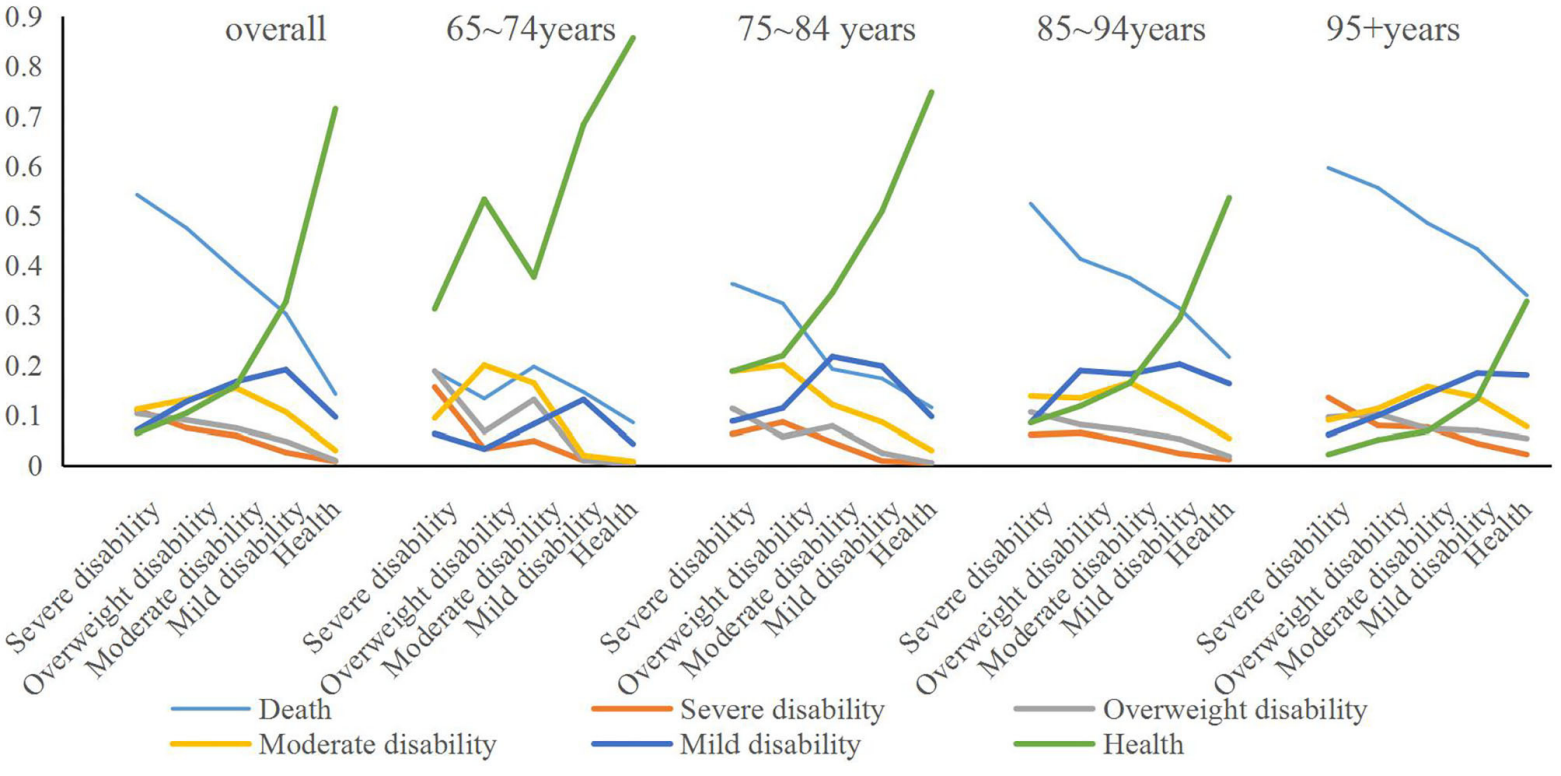
Disability state transfer chain under different age samples.

According to the calculation of the death probability model and the disability state transition probability model, the death state is the absorption state, the age group and the initial health state in 2011 are based on the analysis of the Markov transition probability matrix method, and the Matlab software is used to estimate each. The transition probability in the state and the transition probability matrix are obtained, as shown in Figure 1. The first column of Figure 1 represents each initial disability of the elderly in the matrix in 2011. The initial death status is not listed here, and the first row represents the direction of the elderly's state transition after 3 years, and the sum is equal to 1, which includes the absorption state of death. First of all, from an overall point of view, the elderly with severe disability in the initial state have a 54.2% probability of dying after 3 years, and those who still maintain severe disability are 11.0%, shifting to the difference between severe disability and moderate disability. There were 10.3 and 11.2%, only 7% were transferred to mild disability, and 6.4% were transferred to health. In 2011, it was the elderly with severe incapacitation, and the proportion converted to health in 2014 was 10.4%. Only 7.4% still maintained the severe incapacitation, and the mortality rate was 47.5%. The initial state of the elderly is moderately disabled. In 2014, 15.9% of them became healthy, 15.4% of them remained in their original state, and the mortality rate reached 38.7%. The elderly people with mild disability were initially transferred to health in 2014, reaching 32.7%, those who maintained the original state were 19.1%, and deaths reached 30.3%. The initial state of healthy elderly is 14.2%, and the probabilities of transferring to severe disability, partial disability, severe disability, and mild disability are 0.8, 1.0, 3.0, and 9.6%, respectively. Those who maintained their health comprised 71.5%.

The calculation results by age group show that in the 65–74 year-old age group, the elderly were in the initial state of health, the probability of death was 8.5 and 4.3% were transferred to mild disability; the elderly with mild disability initially maintained 13.1% in their original condition, 68.3% were transferred to health, and 14.6% died; and 16.4% of those with initial moderate disability remained in 2014. The rate of transfer to health was 37.7%, the rate of death was 19.7%, and the probability of transfer to severe disability and partial disability was 4.9 and 13.1%, respectively. The initial state is severely disabled and elderly with severe disability. The mortality rate is 13.3 and 18.8%, respectively, and the probability of becoming healthy is 53.3 and 31.3%, respectively. Among them, the initial state is severely disabled and in 3 years. Later, the sum of maintaining the state of severe disability and partial severe disability reached 34.4%.

In the age group of 75–84 years, the initial healthy elderly have the highest probability of maintaining health in 3 years, reaching 74.8%, while the elderly with severe initial disability have the lowest health transfer probability, only 18.8%, which is the highest mortality rate. It is also the same for the initial severely disabled elderly, reaching 36.3%, and the initial healthy elderly has the lowest probability of death of 21.6%. In terms of other states, the initial state is the elderly with severe disability and partial severe disability, and the probability of being still in the state of partial severe and severe disability during the 3 year period is the highest, while the initial state is the elderly with moderate disability and mild disability. The probability of 3 year transfer is still moderate and mild disability.

In the age group of 85–94 years, the initially healthy elderly have the highest probability of maintaining health in 3 years, reaching 53.6%, while the elderly with severe initial disability have the lowest health transfer probability, only 8.5%, which is the highest mortality rate. It is also the same for the initially severely disabled elderly, reaching 52.4%, and the initial healthy elderly death probability is 11.5%. In terms of other states, the elderly with severe disability in the initial state have the highest probability of being in severe disability during the 3 year period; the elderly with severe, moderate, and mild disability in the initial state. The probability of 3 year transfer is moderate and mild disability.

Under the age of 95+ years (including 95 years old), the initial healthy elderly has the highest probability of maintaining health in 3 years, but it is also only 32.8%, while the elderly with initial severe disability has the lowest health transfer probability, only 2.2%, which is the highest mortality rate. It is also the same for the initial severely disabled elderly, reaching 59.6%, and the initial healthy elderly death probability also reached 34.0%. In other states, the elderly with moderate and mild disability in the initial state have a higher probability of transferring to moderate and mild disability in the 3 year period; the same for the elderly with severe disability in the initial state, and the 3 year period. The probability of being in a state of severe disability is the highest, and the initial state is the elderly with partial severe disability. In addition to maintaining a higher original state in the 3 year state transition, the difference in the probability of its transfer to other disability states is smaller, but it is more. Favored state transfer is better; while the elderly in the initial state are healthy, the probability of transferring to mild and moderate disability in 3 years is higher.

The above results indicate that, overall, the 3 year transfer probability of the initially healthy elderly is the highest, and the mortality rate is also the lowest, while the initial state of severely disabled elderly has the highest mortality rate, which maintains severe disability and transfer. The probability of reaching a disability such as overweight is also the highest. The results of different age groups show that the transition probabilities of old people in different age groups are different, but there is a clear trend of change. Among them, young elderly people aged 65–74, regardless of the initial state of disability, the overall three. The probability of transferring to health in years is the highest, both higher than other age groups, and the mortality rate is generally lower than other age groups. Secondly, the initial state at different ages is the elderly with severe disability and partial disability. The mortality rate is much higher than that of the elderly in other states, and the state of maintaining severe disability or partial disability is also higher.

## Prediction of the Scale of Disabled Population and Multistate Life Expectancy

### Forecast of the Scale of Disabled Population

**Figure 2:**
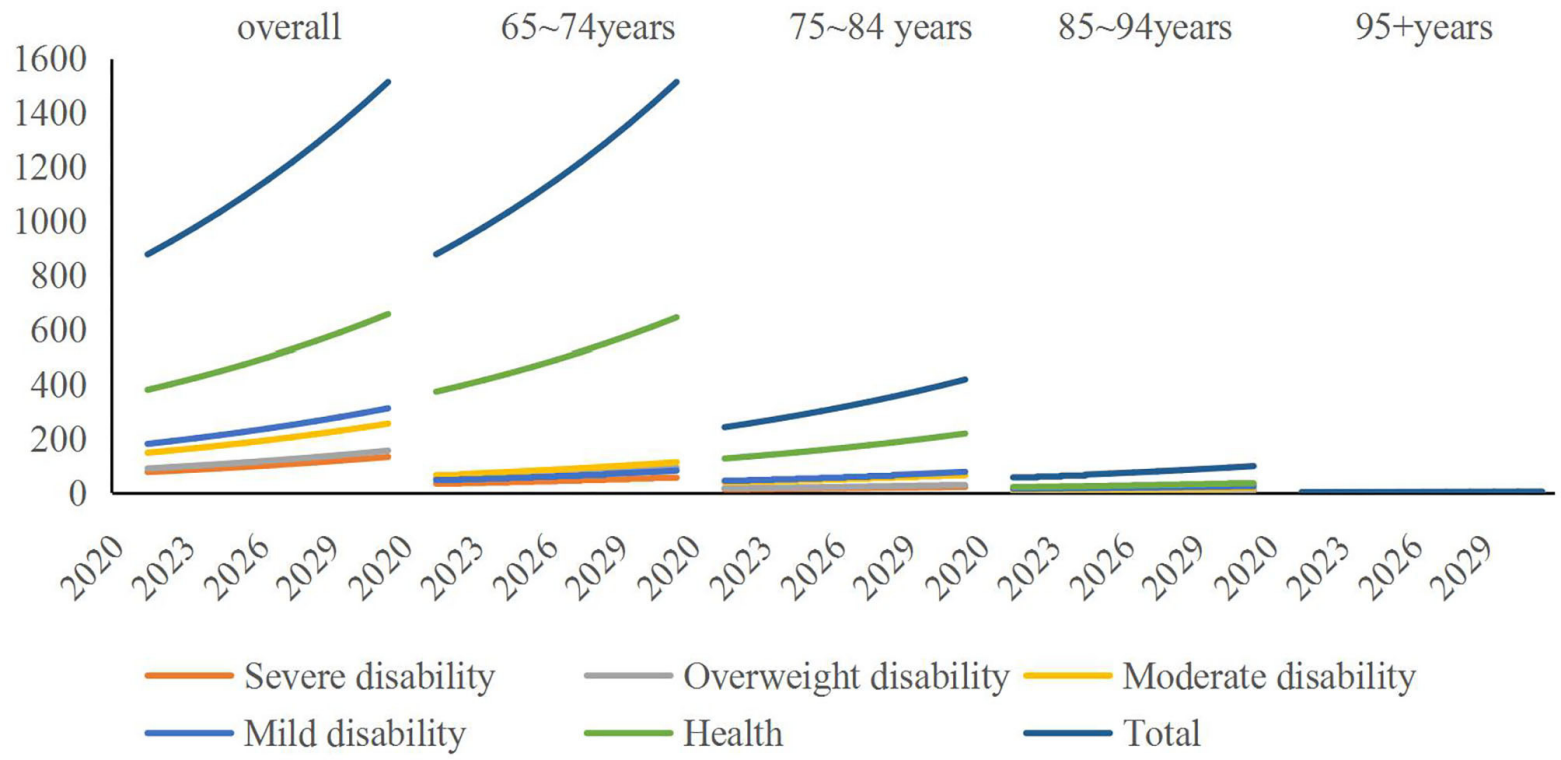
Prediction of total disabled population in Zhejiang Province by age. Unit is 10,000 people.

According to the statistics of the Zhejiang Statistical Yearbook and Chinese Population Yearbook, we selected statistical indicators resident population and registered residence population and 65+ population in Zhejiang Province in the recent 4 years, which is in 2016 ~ 2019. Based on the 4 year statistics of relevant indicators, this paper uses the moving average method to show the predicted values of relevant statistical indicators in Zhejiang Province in the next 10 years. With the help of the above calculation of the transition probability of disabled state, we can estimate the grading and scale of the disabled population in Zhejiang Province at the beginning of the period. The results are shown in Figure 2, and the detailed data are shown in the Tables 1–3 in Appendix. Figure 2 shows the total size of the disabled population in Zhejiang Province in the next 10 years and the size of disabled population in different age groups. The results showed that the total scale of severe disability was the smallest, and the absolute number of severe disability in the 65–74 year-old group was the largest. In addition, with the increase of age, the proportion of severe disability in the total number of disabled elderly is changing, which means that with the increase of age, the proportion of healthy elderly is shrinking, and the difference between different disabled elderly is decreasing, and the main disability is moderate or above, especially in the 95+ age group.

### Multistate Average Life Expectancy

The results in Table 4 show that in the estimation of the duration of total disability by age group, the average life expectancy of the elderly aged 65–74 years reached the highest which is 14.5 years, of which 8.6 years were healthy and severely disabled 0.9 years, about 1 year for severe disability, 1.6 years for moderate disability, and 2.5 years for mild disability; the proportion of multistate disability duration reached 41.16%, indicating that the longer period of time was in multistate—disabled state. The average life expectancy of the elderly aged 75–85 years is 9.3 years, of which the health duration is 4.6 years, and the duration of multistate disability has reached 50.56%, indicating that more than half of the time is in disability. The average life expectancy of the elderly in the 85–95 age group is 7.6 years, of which the health maintenance time is 2.6 years, and the duration of multistate disability reaches 65.78%. The average remaining life of the elderly in the 95+ age group is 5.7 years, of which the health maintenance time is only about 1 year, and the proportion of the duration of multistate disability reaches 81.76%.

**Table 4:** Duration of disability and average life expectancy by age group.

**Age**	**Disabled state duration**					**Average remaining life (years)**
	**Severe disability**	**Overweight disability**	**Moderate disability**	**Mild disability**	**Health**	
65 ~ 74	0.855	0.959	1.642	2.530	8.559	14.545
75 ~ 85	0.686	0.775	1.318	1.949	4.622	9.349
85 ~ 95	0.804	0.894	1.417	1.869	2.592	7.575
95+	0.974	0.997	1.326	1.393	1.046	5.736

The above results show that from different ages, the average remaining life of the youngest, 65–74 years old, is the highest, and the absolute value of the duration of their disability is the largest. With the increase of age, the remaining life of the elderly is decreasing, while the disability is decreasing. The ratio of state maintenance time to life expectancy is also increasing; that is, elderly people face a higher risk of disability and therefore require more nursing services.

Secondly, with the help of the transition probability matrix and the transition strength, the duration of each state of the elderly at different ages and the estimated life expectancy are predicted according to the initial disability grading status. In the 65–74 age group, if the initial state is healthy, the life expectancy is 8.8 years. Among them, the health maintenance time is 7.2 years, the mild disability maintenance time is about 1.1 years, and the severe disability maintenance time is about 0.1 years. Furthermore, the maintenance time in the state of partial and moderate disability is about 0.1 and 0.3 years, respectively. With the improvement of the initial disability level, the average life expectancy is declining, and the elderly with severe disability in the initial state have an average life expectancy of only 0.5 years, which mainly stays in severe disability, severe disability, and moderate disability.

**Figure 3:**
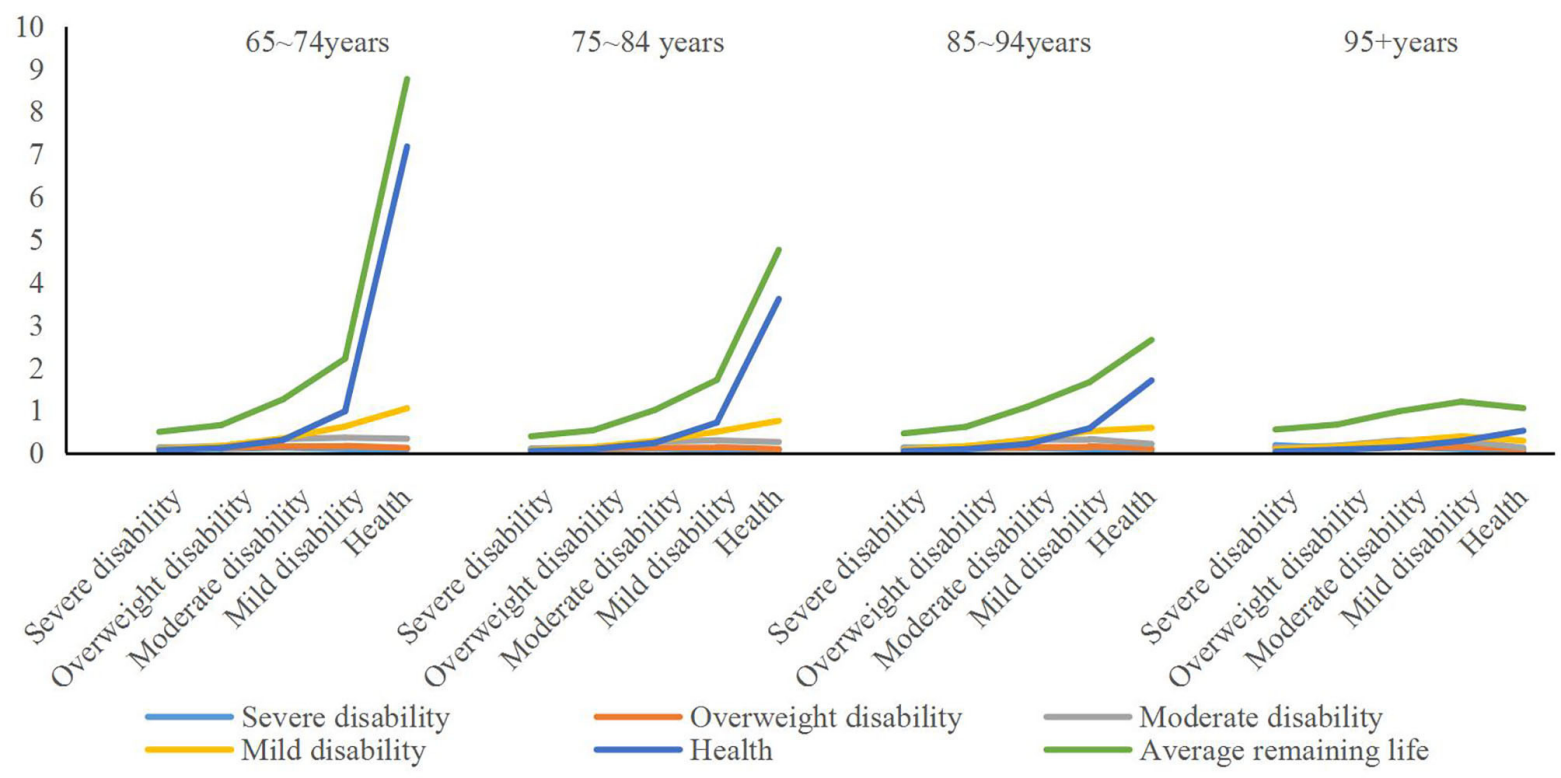
Comparison of the state duration and expected remaining life trend under different age samples. Unit is year.

Moreover, the comparison of the state duration and expected remaining life trends under different age samples is shown in Figure 3. It can also be seen from the results in Figure 3 that in different age groups, the average life expectancy of the elderly between 75–84 years old, 85–94 years old, and 95+ years old during initial health is continuously decreasing, from an average of 4.8. The year has been reduced to 1.1 years, and in the same age group under different age groups, with the improvement of the level of disability, its average remaining life has shown a significant downward trend. However, among the different age groups, other elderly people with initial disability did not show a clear trend of age, especially when the initial state was severe disability and partial disability, in the 85–94-year-old and 95+ age groups. The elderly have a higher average life expectancy and stay in the state above moderate disability for much longer than other age groups.

The purpose of predicting the duration of multistate disability and the average life expectancy is to investigate the length of time that different elderly people with disability need care after disability during their lifetime. It can be seen from the results in Figure 3 that when considering the absolute amount, the care needs of the elderly with moderate or above disability at different ages are higher, but there is a clear linear trend between different age groups. At different ages, the elderly with an initial state of moderate to higher levels of disability have a higher average demand for disability care during their lifetime, and the strength of care needs under severe and severe disability.

## Discussion

The article builds a multistate disability index system based on multiple dimensions and ranks the disabled elderly. Based on the current domestic research on the definition of disability, it can be found that most scholars mainly rely on the experience of early disability definition, such as the definition of six basic functional areas (ADLs). In recent years, scholars have drawn on the introduction of foreign scholars and official research or experience on the definition of disability, combined basic functional areas with instrumental activities of daily living (IADLs) to study the state of disability, and gradually bring cognitive function into the study. However, by combining the existing research, it is found that although the existing research considers three scales of different scales for measurement, they do not consider the difference in index weights of different dimensions. For example, although the research of Huang and Wu is also based on the consideration of three dimensions (32), it ignores the difference in the index assignment of different dimensions, and only a parallel way of inspection will cause the irrationality of the evaluation results to a certain extent, making it unreasonable. Reliability can be assessed in stages.

Based on the existing research, combined with the characteristics of China's current long-term care social insurance pilot areas, it can be found that the definition of the basic disability level population is an important basis for promoting the development of long-term care insurance or long-term care services. Therefore, the article attempts to base on the basic functions of ICF. The domain framework examines the definition of the disability level of the elderly from multiple dimensions and draws on existing research experience to try to divide the disability level into five levels and assign specific weights for each dimension through the secondary index scores, which is better in sorting out the differences in the intensity of care needs of different aged people. The research results show that, compared with the existing disability definition, this disability definition has better reliability. First, the disability rating is reasonable. The significance of conducting disability assessment is not only to effectively distinguish elderly people with different levels of disability, but the most fundamental purpose is to accurately characterize the disability status and characteristics of elderly people with different levels of disability, and then to identify nursing needs and provide service provision at different levels, such as basic support. Disability level division is the basis for the division of nursing graded service supply. After the assessment of disability, elderly people of different levels are provided with nursing service content according to the level of disability (33). In addition, the reasonable disability level division helps to identify the dynamic changes of the physical conditions of the elderly at different ages and adjust the care plan in time. The article is based on the ICF framework and comprehensively considers the functional areas of disability assessment at different ages. Through the disability grading and result calculation, it can effectively reflect the comprehensive level of various functions of the elderly, and the entries are simplified. Secondly, on the basis of the existing research, the different disability levels within the disability grading in this paper are different and the division is reasonable. At present, most of the pilot areas in China use the Barthel Scale as a tool for long-term care insurance evaluation and use severe disability (≤40 points) as the boundary of insurance payments to give priority to the protection of severely disabled people (5, 34). This article collects the four dimensions of restricted activity, physical function and structural damage, restricted participation, and health. When the disability level is divided, there is an obvious difference in the score interval, indicating that its function declines as the comprehensive level decreases, and it manifests in multiple aspects, which has a greater impact on disability.

## Conclusion

Based on the ICF functional framework, the article uses CLHLS database 2011 and 2014 tracking survey data to construct a multidimensional disability index system and obtains corresponding scores for each dimension according to the assignment of different items and then performs disability grading. On the basis, it constructs the probability model of death and the probability model of disability transition and empirically tests the effectiveness of disability grading. The empirical test results show that the four dimensions of activity limitation, physical function and structural damage, participation limitation, and health are important influencing factors for mortality of elderly people of different ages, and the four dimensions are the core explanatory variables for the transfer of disability. The four threshold parameters under the logit model are all significant, indicating that the selection of disability grading index is reasonable and effective.

Through the construction of multistate disability-grading indicators, based on 2011 and 2014 tracking survey data, based on Markov chain, this article calculated the multistate disability transition probability, constructed a 3 year disability state transition probability matrix, and divided it into population and age. The results show that the 3 year transfer probability of the initial healthy elderly is the highest, and the mortality rate is also the lowest, while the initial state of the elderly is severely disabled, the mortality rate is the highest, and the maintenance of severe incapacitation and the shift to partial severe disability probability of energy state is also the highest. Next, the initial state at different ages is the elderly with severe disability and partial disability. The mortality rate is much higher than that of the elderly in other states, and the state of maintaining severe disability or partial disability is also higher. From different ages, the average remaining life of 65–74 is the highest, and the absolute value of the duration of their disability is the largest. With the increase of age, the remaining life of the elderly in the elderly is decreasing, while the disability maintains the life-span ratio which is also increasing, which means that the elderly are facing a higher risk of disability and therefore require more nursing services.

The conclusion and enlightenment of this paper is as follows: Firstly, in the design process of independent insurance of long-term care social insurance, we should first clarify the grading standard of disabled objects, highlight the essential characteristics of “basic insurance” of social insurance, and focus on the realization of basic care and nursing needs satisfaction of people with the highest demand intensity. Secondly, in order to avoid the phenomenon of “upside down” between payment and treatment, we should build a financing system based on the characteristics of social insurance and appropriate consideration of social welfare, such as the establishment of differentiated rate financing mechanism by age based on disability grading. Moreover, we should follow the concept of phased development in the scope of protection and gradually expand the scope of benefit by taking the severely disabled objects as the main body in the initial stage, and the differences in the protection standards or levels of the beneficiary groups under different disability levels should be appropriate, so as to avoid excessive adverse selection and moral hazard.

## Data Availability Statement

The datasets presented in this study can be found in online repositories. The names of the repository/repositories and accession number(s) can be found at: https://opendata.pku.edu.cn/dataset.xhtml?persistentId=doi:10.18170/DVN/XRV2WN.

## Author Contributions

HL revised it critically for important intellectual content and approved the version to be published, carry out language retouching, and modification. HL made a substantial contribution to the concept and design of the work, interpretation of data, and drafted the article.

## Conflict of Interest

The author declares that the research was conducted in the absence of any commercial or financial relationships that could be construed as a potential conflict of interest.
