# Supplementary material for: Research on Disability Grading Based on ICF Functional Framework: Empirical Evidence From Zhejiang Province, China
Source: Front Public Health. 2021 May 11;9:616180. doi: 10.3389/fpubh.2021.616180 (PMC8144326; doi:10.3389/fpubh.2021.616180)
Supplement: Supplementary file 1 [file Data_Sheet_1.docx]

**Appendix：**

Table 1 Probability of disability transition during three years by age group

| 2011 status | 2014 health status | | | | | |
| --- | --- | --- | --- | --- | --- | --- |
|  | Death | Severe disability | Overweight disability | Moderate disability | Mild disability | Health |
| overall | | | | | | |
| Severe disability | 0.542 | 0.110 | 0.103 | 0.112 | 0.070 | 0.064 |
| Overweight disability | 0.475 | 0.074 | 0.090 | 0.131 | 0.127 | 0.104 |
| Moderate disability | 0.387 | 0.059 | 0.074 | 0.154 | 0.167 | 0.159 |
| Mild disability | 0.303 | 0.026 | 0.048 | 0.106 | 0.191 | 0.327 |
| Health | 0.142 | 0.008 | 0.010 | 0.030 | 0.096 | 0.715 |
| Age group 65~75 years old | | | | | | |
| Severe disability | 0.188 | 0.156 | 0.188 | 0.094 | 0.063 | 0.313 |
| Overweight disability | 0.133 | 0.033 | 0.067 | 0.200 | 0.033 | 0.533 |
| Moderate disability | 0.197 | 0.049 | 0.131 | 0.164 | 0.082 | 0.377 |
| Mild disability | 0.146 | 0.010 | 0.010 | 0.020 | 0.131 | 0.683 |
| Health | 0.085 | 0.005 | 0.003 | 0.008 | 0.043 | 0.857 |
| Age group 75~85 years old | | | | | | |
| Severe disability | 0.363 | 0.063 | 0.113 | 0.188 | 0.088 | 0.188 |
| Overweight disability | 0.324 | 0.086 | 0.057 | 0.200 | 0.114 | 0.219 |
| Moderate disability | 0.192 | 0.046 | 0.078 | 0.121 | 0.217 | 0.345 |
| Mild disability | 0.173 | 0.009 | 0.025 | 0.086 | 0.198 | 0.509 |
| Health | 0.115 | 0.005 | 0.005 | 0.030 | 0.097 | 0.748 |
| Age group 85~95years old | | | | | | |
| Severe disability | 0.524 | 0.061 | 0.106 | 0.138 | 0.085 | 0.085 |
| Overweight disability | 0.413 | 0.065 | 0.081 | 0.134 | 0.189 | 0.118 |
| Moderate disability | 0.375 | 0.046 | 0.069 | 0.165 | 0.182 | 0.164 |
| Mild disability | 0.314 | 0.024 | 0.053 | 0.112 | 0.202 | 0.294 |
| Health | 0.216 | 0.012 | 0.018 | 0.054 | 0.163 | 0.536 |
| Age group 95+years old | | | | | | |
| Severe disability | 0.596 | 0.135 | 0.096 | 0.090 | 0.061 | 0.022 |
| Overweight disability | 0.556 | 0.079 | 0.102 | 0.113 | 0.099 | 0.051 |
| Moderate disability | 0.485 | 0.076 | 0.073 | 0.157 | 0.142 | 0.068 |
| Mild disability | 0.433 | 0.044 | 0.069 | 0.136 | 0.184 | 0.133 |
| Health | 0.340 | 0.022 | 0.054 | 0.077 | 0.180 | 0.328 |

Table 2 Prediction of total disabled population in Zhejiang Province by age group

(Unit: ten thousand people)

| Age group | Severe disability | Overweight disability | Moderate disability | Mild disability | Health | Total |
| --- | --- | --- | --- | --- | --- | --- |
| Total disabled population size | | | | | | |
| 2020 | 77.0269 | 90.3619 | 148.2637 | 181.0747 | 380.7482 | 877.4755 |
| 2021 | 81.3485 | 95.4316 | 156.5819 | 191.2337 | 402.1097 | 926.7053 |
| 2022 | 85.9123 | 100.7855 | 165.3665 | 201.9624 | 424.6690 | 978.6957 |
| 2023 | 90.7316 | 106.4392 | 174.6429 | 213.2917 | 448.4913 | 1033.5967 |
| 2024 | 95.8214 | 112.4101 | 184.4398 | 225.2567 | 473.6502 | 1091.5783 |
| 2025 | 101.1974 | 118.7168 | 194.7877 | 237.8946 | 500.2241 | 1152.8205 |
| 2026 | 106.8746 | 125.3768 | 205.7153 | 251.2404 | 528.2865 | 1217.4935 |
| 2007 | 112.8704 | 132.4106 | 217.2563 | 265.3355 | 557.9244 | 1285.7971 |
| 2028 | 119.2025 | 139.8390 | 229.4445 | 280.2210 | 589.2244 | 1357.9315 |
| 2029 | 125.8894 | 147.6835 | 242.3156 | 295.9404 | 622.2779 | 1434.1068 |
| 2030 | 132.9520 | 155.9688 | 255.9099 | 312.5433 | 657.1888 | 1514.5629 |
| Age group 65~74 years old | | | | | | |
| 2020 | 34.2321 | 53.9660 | 65.7602 | 47.6373 | 373.7919 | 575.3875 |
| 2021 | 36.1528 | 56.9938 | 69.4498 | 50.3101 | 394.7642 | 607.6707 |
| 2022 | 38.1812 | 60.1915 | 73.3463 | 53.1328 | 416.9125 | 641.7643 |
| 2023 | 40.3232 | 63.5683 | 77.4611 | 56.1136 | 440.3017 | 677.7679 |
| 2024 | 42.5847 | 67.1336 | 81.8057 | 59.2608 | 464.9968 | 715.7816 |
| 2025 | 44.9743 | 70.9006 | 86.3959 | 62.5860 | 491.0887 | 755.9455 |
| 2026 | 47.4971 | 74.8777 | 91.2423 | 66.0968 | 518.6360 | 798.3499 |
| 2007 | 50.1621 | 79.0790 | 96.3618 | 69.8054 | 547.7361 | 843.1444 |
| 2028 | 52.9758 | 83.5148 | 101.7670 | 73.7210 | 578.4604 | 890.439 |
| 2029 | 55.9479 | 88.2001 | 107.4763 | 77.8568 | 610.9129 | 940.394 |
| 2030 | 59.0865 | 93.1481 | 113.5056 | 82.2245 | 645.1846 | 993.1493 |
| Age group 75~84years old | | | | | | |
| 2020 | 13.2202 | 17.5866 | 39.5395 | 45.1672 | 127.0842 | 242.5977 |
| 2021 | 13.9620 | 18.5733 | 41.7578 | 47.7012 | 134.2142 | 256.2085 |
| 2022 | 14.7453 | 19.6153 | 44.1005 | 50.3774 | 141.7439 | 270.5824 |
| 2023 | 15.5724 | 20.7156 | 46.5744 | 53.2034 | 149.6952 | 285.761 |
| 2024 | 16.4460 | 21.8777 | 49.1870 | 56.1879 | 158.0926 | 301.7912 |
| 2025 | 17.3687 | 23.1051 | 51.9467 | 59.3403 | 166.9623 | 318.7231 |
| 2026 | 18.3430 | 24.4013 | 54.8608 | 62.6693 | 176.3288 | 336.6032 |
| 2007 | 19.3721 | 25.7703 | 57.9386 | 66.1851 | 186.2212 | 355.4873 |
| 2028 | 20.4589 | 27.2160 | 61.1891 | 69.8982 | 196.6684 | 375.4306 |
| 2029 | 21.6066 | 28.7427 | 64.6216 | 73.8192 | 207.7008 | 396.4909 |
| 2030 | 22.8188 | 30.3552 | 68.2469 | 77.9606 | 219.3532 | 418.7347 |
| Age group 85~94years old | | | | | | |
| 2020 | 3.7810 | 5.9441 | 10.9644 | 14.9233 | 21.7625 | 57.3753 |
| 2021 | 3.9932 | 6.2776 | 11.5796 | 15.7606 | 22.9835 | 60.5945 |
| 2022 | 4.2172 | 6.6298 | 12.2292 | 16.6448 | 24.2729 | 63.9939 |
| 2023 | 4.4538 | 7.0017 | 12.9152 | 17.5785 | 25.6345 | 67.5837 |
| 2024 | 4.7036 | 7.3944 | 13.6400 | 18.5646 | 27.0725 | 71.3751 |
| 2025 | 4.9675 | 7.8093 | 14.4050 | 19.6062 | 28.5914 | 75.3794 |
| 2026 | 5.2462 | 8.2474 | 15.2131 | 20.7061 | 30.1954 | 79.6082 |
| 2007 | 5.5405 | 8.7101 | 16.0666 | 21.8677 | 31.8894 | 84.0743 |
| 2028 | 5.8513 | 9.1987 | 16.9680 | 23.0945 | 33.6784 | 88.7909 |
| 2029 | 6.1796 | 9.7148 | 17.9198 | 24.3901 | 35.5677 | 93.772 |
| 2030 | 6.5262 | 10.2598 | 18.9251 | 25.7584 | 37.5631 | 99.0326 |
| Age group 95+ years old | | | | | | |
| 2020 | 0.4098 | 0.4537 | 0.6598 | 0.7666 | 0.6929 | 2.9828 |
| 2021 | 0.4328 | 0.4791 | 0.6968 | 0.8096 | 0.7318 | 3.1501 |
| 2022 | 0.4571 | 0.5060 | 0.7359 | 0.8550 | 0.7728 | 3.3268 |
| 2023 | 0.4828 | 0.5344 | 0.7772 | 0.9030 | 0.8162 | 3.5136 |
| 2024 | 0.5098 | 0.5644 | 0.8208 | 0.9536 | 0.8620 | 3.7106 |
| 2025 | 0.5384 | 0.5961 | 0.8668 | 1.0071 | 0.9103 | 3.9187 |
| 2026 | 0.5687 | 0.6295 | 0.9155 | 1.0636 | 0.9614 | 4.1387 |
| 2007 | 0.6006 | 0.6648 | 0.9668 | 1.1233 | 1.0153 | 4.3708 |
| 2028 | 0.6342 | 0.7021 | 1.0211 | 1.1863 | 1.0723 | 4.616 |
| 2029 | 0.6698 | 0.7415 | 1.0783 | 1.2529 | 1.1325 | 4.875 |
| 2030 | 0.7074 | 0.7831 | 1.1388 | 1.3232 | 1.1960 | 5.1485 |

Table 3 State duration and average life expectancy of different age groups according to the state transition of disability classification

| 2011 status | 2014 health status | | | | | | | | Average remaining life |
| --- | --- | --- | --- | --- | --- | --- | --- | --- | --- |
|  | Severe disability | | Overweight disability | Moderate disability | | Mild disability | | Health |  |
| Age group 65~75 years old | | | | | | | | | |
| Severe disability | 0.124 | 0.112 | | 0.119 | 0.076 | | 0.062 | | 0.494 |
| Overweight disability | 0.096 | 0.118 | | 0.163 | 0.159 | | 0.116 | | 0.652 |
| Moderate disability | 0.127 | 0.155 | | 0.326 | 0.344 | | 0.305 | | 1.257 |
| Mild disability | 0.089 | 0.165 | | 0.358 | 0.621 | | 0.978 | | 2.211 |
| Health | 0.083 | 0.118 | | 0.333 | 1.048 | | 7.179 | | 8.760 |
| Age group 75~85 years old | | | | | | | | | |
| Severe disability | 0.099 | 0.089 | | 0.097 | 0.061 | | 0.044 | | 0.390 |
| Overweight disability | 0.079 | 0.097 | | 0.132 | 0.132 | | 0.088 | | 0.529 |
| Moderate disability | 0.103 | 0.122 | | 0.264 | 0.282 | | 0.236 | | 1.007 |
| Mild disability | 0.072 | 0.136 | | 0.295 | 0.497 | | 0.712 | | 1.711 |
| Health | 0.055 | 0.090 | | 0.255 | 0.753 | | 3.607 | | 4.760 |
| Age group 85~95 years old | | | | | | | | | |
| Severe disability | 0.124 | 0.107 | | 0.112 | 0.073 | | 0.043 | | 0.457 |
| Overweight disability | 0.093 | 0.119 | | 0.150 | 0.159 | | 0.090 | | 0.611 |
| Moderate disability | 0.121 | 0.137 | | 0.309 | 0.307 | | 0.211 | | 1.085 |
| Mild disability | 0.088 | 0.159 | | 0.324 | 0.510 | | 0.578 | | 1.659 |
| Health | 0.051 | 0.096 | | 0.211 | 0.590 | | 1.700 | | 2.649 |
| Age group95+ years old | | | | | | | | | |
| Severe disability | 0.183 | 0.130 | | 0.122 | 0.083 | | 0.030 | | 0.548 |
| Overweight disability | 0.119 | 0.153 | | 0.170 | 0.149 | | 0.077 | | 0.666 |
| Moderate disability | 0.144 | 0.138 | | 0.297 | 0.269 | | 0.129 | | 0.977 |
| Mild disability | 0.093 | 0.146 | | 0.289 | 0.390 | | 0.282 | | 1.201 |
| Health | 0.035 | 0.086 | | 0.122 | 0.286 | | 0.521 | | 1.050 |

***Disability transition probability and transition intensity***

In terms of the trajectory of the disability elderly, by constructing a dynamic transition probability model of the disability state, the article analyzes the state transition matrix of the disability elderly and evaluates the net increase. Comprehensively evaluate the size and evolution of the disabled elderly in the future, and use the multi-state Markov chain non-homogeneous assumption to introduce the transition strength as a piecewise constant, and use the Markov method to multiply the age cohort matrix to predict the number of graded disability people.

Firstly, the disability state transition probability matrix is estimated. Multi-state life tables are generally estimated based on population or state. Remember$P_{ij}(x)$（$i,j=1,2,\cdots,N$）is the probability that a person who is in a state$i$at the age$x$will be in a state$j$at the age$x+n$. Therefore, the state transition probability matrix for each age range can be written as

（1）

Define the rate of state$m_{ij}(x)$transition from state$i$to state$j$as the number of people who transitioned from state$i$ to age$(x,x+n)$, divided by the number of years in the same age group that survived in the state$i$. Remember the mortality rate$m_{i}(x)$in the state$i$as the combination of the transition probabilities of each state and the mortality rate into a special matrix$m(x)$, namely formula (2):

（2）

The formula for estimating the multi-state transition probability matrix based on the multi-state transition probability matrix is similar to the formula for estimating the survival rate based on the mortality, that

$P(x)=[I+n/2*m(x)]^{-1}[I-n/2*m(x)]$ or $P(x)=\exp(-nm(x))$ （3）

Among them, the identity matrix is in the formula. The multi-state life table starts at the age $y$and is recorded${}_{iy}l(x)$ as the number$j$of people who were in the state$i$at the age$y$and survived at the age$x$. According to Markov's assumption about state transition, that the probability of state transition from age r$x$to$x+n$depends only on the state at the age$x$, and has nothing to do with the state before the year of age$x$, we can get:

（4）

Among them,$i,j,k=1,2,3,\cdots,N$is the total number of states. Its matrix form is

（5）

Secondly, the strength of transition and state duration are estimated. In the multi-state life table, it is recorded${}_{iy}L(x)$as the number of years of survivors who are in a state$i$of age$y$and in a state$j$ of age$(x,x+n)$. It is recorded${}_{iy}T(x)$as the number of survivors in the state$j$above the age$x$when they are$y$in the state$i$. The article attempts to measure the transfer intensity based on the number of survivors and years of survivors after 3 years, that is, the multi-state transfer intensity of this paper is based on a three-year period.
